# Association of Statin Use With Cancer- and Noncancer-Associated Survival Among Patients With Breast Cancer in Asia

**DOI:** 10.1001/jamanetworkopen.2023.9515

**Published:** 2023-04-21

**Authors:** Wei-Ting Chang, Hui-Wen Lin, Sheng-Hsiang Lin, Yi-Heng Li

**Affiliations:** 1 Institute of Clinical Medicine, College of Medicine, National Cheng Kung University, Tainan, Taiwan; 2 Division of Cardiology, Department of Internal Medicine, Chi-Mei Medical Center, Tainan, Taiwan; 3 Department of Biotechnology, Southern Taiwan University of Science and Technology, Tainan, Taiwan; 4 Department of Internal Medicine, National Cheng Kung University Hospital, College of Medicine, National Cheng Kung University, Tainan, Taiwan; 5 Biostatistics Consulting Center, National Cheng Kung University Hospital, College of Medicine, National Cheng Kung University, Tainan, Taiwan; 6 Department of Public Health, College of Medicine, National Cheng Kung University, Tainan, Taiwan.

## Abstract

**Importance:**

In addition to protective effects on the cardiovascular system, statins may reduce the risk of breast cancer recurrence owing to potential anti-inflammatory benefits. Given that patients with breast cancer in Asia are relatively younger at diagnosis and most are free from traditional cardiovascular risk factors, it is uncertain whether the use of statins can improve survival.

**Objective:**

To investigate the association of statin use with cancer- and noncancer-associated survival in patients with breast cancer.

**Design, Setting and Participants:**

This cohort study used the Taiwanese National Health Insurance Research Database and National Cancer Registry to identify patients diagnosed with breast cancer from January 2012 to December 2017. Age, cancer stage, anticancer therapies, comorbidities, socioeconomic status, and cardiovascular drugs were matched by propensity score method. Statistical analyses, including Cox proportional hazards models, were performed from June 2022 to February 2023. The mean (SD) follow-up duration was 4.10 (2.96) years.

**Interventions:**

Patients receiving statins within 6 months before the diagnosis of breast cancer were compared with those not receiving statins.

**Main Outcomes and Measures:**

Outcomes included death, heart failure, and arterial and venous events.

**Results:**

Overall, 7451 patients (mean [SD] age, 64.3 [9.4] years) treated with statins were matched with 7451 nonusers (mean [SD] age, 65.8 [10.8] years). Compared with nonusers, statin users had a significantly lower risk of all-cause death (adjusted hazard ratio [HR], 0.83; 95% CI, 0.77-0.91; *P* < .001). Notably, the risk reduction was mainly attributed to cancer-related death (adjusted HR, 0.83; 95% CI, 0.75-0.92; *P* < .001). Only a small number of patients died of cardiovascular causes, and the ratios were similar between statin users and nonusers. No significant differences were observed in cardiovascular outcomes, including heart failure and arterial and venous events, between statin users and nonusers. Using a time-dependent analysis, statin users also presented a significantly lower risk of cancer-related death (adjusted HR, 0.28; 95% CI, 0.24-0.32; *P* < .001) than nonusers, and notably, the risk was even lower in high-dose statin (HDS) users compared with non-HDS users (HDS users: adjusted HR, 0.84; 95% CI, 0.73-0.98; *P* = .002; non-HDS users: adjusted HR, 0.79; 95% CI, 0.68-0.91; *P* = 001).

**Conclusions and Relevance:**

In this cohort study of Asian patients with breast cancer, statin use was associated with a reduced risk of cancer-associated death rather than cardiovascular death. Our findings provide evidence to support the use of statins in patients with breast cancer; however, randomized studies are necessary.

## Key Points

**Question:**

Is statin use associated with lower cancer- and noncancer-associated death in patients with breast cancer?

**Findings:**

In this cohort study of 14 902 patients with breast cancer, Cox proportional hazards models showed that compared with nonusers, statin users had a significantly lower risk of cancer-related death. The ratios of patients who experienced cardiovascular outcomes, including cardiovascular death, heart failure, and arterial and venous events, were similar between statin users and nonusers after adjustments.

**Meaning:**

These results suggest that statin use was associated with a reduced risk of cancer-related death in patients with breast cancer.

## Teaser

This cohort study investigates the association of statin use with cancer- and noncancer-associated survival among patients in Taiwan with breast cancer.

## Introduction

With the improvement of anticancer therapies, the cancer-free ratio of patients with breast cancer has increased, but survivors face threats of cancer therapy–associated cardiovascular complications, including heart failure (HF) and thrombosis.^1,2^ Through suppressing HMG-CoA reductase, statins have been shown to reduce blood cholesterol, mitigate atherosclerosis, and improve cardiovascular outcomes; however, they have also been observed to have pleiotropic effects on anti-inflammation and antiproliferation in cancer cells.^3,4,5^ Previous studies have found that statins suppress cancer progression and micrometastasis in conjunction with chemotherapy.^4,5^ The anticancer properties of statins have been found in various cancers. In a large Danish cohort, compared with patients who had never used statins, statin users presented with significantly reduced cancer-related deaths for 13 different cancer types, including breast cancer.^6^ Likewise, a retrospective study found that statin use was associated with risk reduction in all subtypes of breast cancer, especially in patients with estrogen receptor–positive and *EBBR2*-negative invasive breast cancer.^7^ This finding implies a plausible link between statin use and outcomes in female patients with cancer. Notably, in contrast to Western patients with breast cancer, Asian patients are relatively younger at diagnosis, and a large proportion are premenopausal and have few cardiovascular risk factors.^8^ Using the Taiwanese National Cohort, our previous work highlighted an increased risk of major adverse cardiovascular and cerebrovascular events, especially HF, in Asian patients receiving trastuzumab.^9^ However, an effective strategy to prevent or rescue anticancer therapy–related cardiovascular complications is lacking. Therefore, this study aimed to explore the association between statin use and cancer- and cardiovascular-associated survival in Asian patients with breast cancer.

## Methods

### Study Design

In this retrospective cohort study, using the Taiwanese National Health Insurance Research Database (NHIRD) and the National Cancer Registry, we identified patients with breast cancer initiating stains from January 2012 to December 2017. This study was approved by the review committee of National Cheung Kung University Hospital, which granted a waiver of informed consent due to the study’s retrospective nature. We followed the Strengthening the Reporting of Observational Studies in Epidemiology (STROBE) reporting guideline.

The index date was set as the diagnosis of breast cancer, and patients who initiated statin use 6 months before the diagnosis were included. Patients who had a previous history of breast cancer, were younger than 18 years, had incomplete data, were male, died before the index date, or had missing data on cancer stage or previous history of outcomes were excluded. The nonexposure group was defined as matched participants without statin use during the same period, and they were matched 1:1 with statin users for the index date variables. Parameters used for propensity score matching included age, cancer stage, anticancer therapies, comorbidities, socioeconomic status, and cardiovascular drugs. To maintain the quality of the data, statin users were required to receive statins more than 90% of the time in the subsequent 30 days during the treatment. Data used in this study were obtained from the original claims database for the reimbursement of all Taiwanese residents from the NHIRD.^10,11^ Information on age, sex, medical history, and concomitant medications or procedures within 3 months after the index date were captured from the database. The diagnostic codes in the NHIRD were identified using the *International Classification of Diseases, Ninth Revision, Clinical Modification* (*ICD-9-CM*) for cases before 2015 and the *International Classification of Diseases, Tenth Revision, Clinical Modification* (*ICD-10-CM*) for cases since 2016. The *ICD* codes are listed in eTable 1 in Supplement 1. It is also feasible to link and continuously follow up on all claims data belonging to the same patient within the NHIRD. Additionally, we identified patients with breast cancer at all stages using the nationwide cancer registration system in Taiwan. All comorbid conditions and corresponding treatments starting a year prior to diagnosis were extracted from the NHIRD as well as medication records of breast cancer diagnosis and treatment. A flowchart of this study is displayed in eFigure 1 in Supplement 1.

### Study End Point

The primary outcome was death. The reasons for death included cardiovascular death, cancer-associated death, and death from other causes. The primary outcomes consisted of new-onset acute HF, arterial events, including acute myocardial infarction and ischemic stroke, and venous events, including deep venous thrombosis (DVT) and pulmonary embolism. Because the *ICD-9-CM* was replaced by *ICD-10-CM* by the Taiwan National Health Insurance in 2016, both editions’ codes (eTable 1 in Supplement 1) were used to identify end points in the primary outcome during follow-up. Patients were followed up from the index date until reaching the outcomes, loss to follow-up, death, or the end of the study period (December 31, 2019). The mean (SD) follow-up duration was 4.10 (2.96) years.

### Statistical Analysis

Continuous variables are presented as mean with SD or median with IQR, and categorical variables are presented as numbers and percentages. Because of the nonrandomized nature of the study, propensity score analysis was performed to minimize any selection bias caused by differences in the clinical characteristics between the groups. The propensity score was defined as the probability of exposure to treatment, conditional on the baseline characteristics of the study participants. In this study, the propensity score for receiving statins was computed using multivariate logistic regression analysis, conditional on covariates including index year, age, cancer stage, anticancer therapies, comorbidities, socioeconomic status, and cardiovascular drugs before enrollment. The distributions of the clinical characteristics in the 2 groups were evaluated using the absolute standardized mean difference (ASMD) rather than statistical testing. The ASMD was calculated as the mean or proportion of a variable divided by the pooled estimate of the SD of that variable, and an ASMD less than 0.1 indicates a negligible difference between the 2 groups. Imbalanced parameters were adjusted. For parameters not fulfilling proportional hazards assumptions, a stratified Cox proportional hazards model was used to examine the association between the end points and different treatments. In this study, we divided the reasons of death into cardiovascular death, cancer-associated death, and death due to other reasons. Considering that death may reduce the incidence of outcomes, the competing risk approach (subdistribution hazard ratio [HR]), following the Fine and Gray method,^12^ was also used to estimate the risk of cancer associated death, HF, and arterial and venous events from the Cox regression model. For cancer or cardiovascular death, the competing risk was adjusted according to other cause of death. For secondary outcomes, including HF and arterial and venous events, the competing risk was adjusted according to all-cause death. In consideration of the effect of time-varying exposure to statins, we performed a time-dependent analysis using statin exposure time as a segmented time-dependent covariate. A Kaplan-Meier curve was constructed for the log-rank test to compare differences between groups. Also, results of the cumulative incidence of cancer death adjusted with competing risk events of other cause of death were also plotted using the Gray test. Furthermore, to estimate the *P* values for interactions in the subgroup analysis, we used the same Cox proportional hazards model. To test the association of different types and doses of statins with the outcomes, we defined patients as high-dose statin (HDS) users (≥10 mg rosuvastatin, ≥20 mg atorvastatin, or ≥40 mg simvastatin at the initial regimens) and non-HDS users and performed subgroup analysis.^13^ The statistical significance was set at *P* < .05, and all tests were 2-tailed. SAS version 9.4 for Windows (SAS Institute) was used for all data analyses. Statistical analyses were performed from June 2022 to February 2023.

## Results

### Demographic Characteristics of Statin Users and Nonusers With Breast Cancer

We initially identified 7777 female patients receiving statins 6 months before they were diagnosed with breast cancer. Compared with 55 753 statin nonusers, we found that cancer stage, anticancer therapies, and socioeconomic status were similar between the 2 groups (Table 1). However, statin users were relatively older and had more coronary artery disease (CAD), hypertension, and diabetes. Additionally, the prevalence of angiotensin-converting enzyme inhibitor/angiotensin receptor blocker (ACEI/ARB) and antiplatelet agent use was relatively higher among patients who received statins vs those who did not (ACEI/ARB: 3670 [47.19%] vs 5798 [10.40%]; antiplatelet: 1634 [21.01%] vs 1729 [3.10%]). After propensity score matching, 7451 statin users (mean [SD] age, 64.33 [9.40] years) were compared with 7451 statin nonusers (mean [SD] age, 65.78 [10.81] years).

**Table 1.** Baseline Characteristics of Statin Users and Nonusers Among Patients With Breast Cancer Before and After Propensity Score Matching

Characteristic	Before propensity score matching				After propensity score matching			
	No. (%)			ASMD	No. (%)			ASMD
	Total (N = 63 530)	Statin users (n = 7777)	Statin nonusers (n = 55 753)		Total (n = 14 902)	Statin users (n = 7451)	Statin nonusers (n = 7451)	
Year								
2012	9221 (14.51)	954 (12.27)	8267 (14.83)	0.15	1876 (12.59)	938 (12.59)	938 (12.59)	0.00
2013	9743 (15.34)	1048 (13.48)	8695 (15.60)		2088 (14.01)	1044 (14.01)	1044 (14.01)	
2014	10 283 (16.19)	1248 (16.05)	9035 (16.21)		2434 (16.33)	1217 (16.33)	1217 (16.33)	
2015	10 874 (17.12)	1323 (17.01)	9551 (17.13)		2522 (16.92)	1261 (16.92)	1261 (16.92)	
2016	10 985 (17.29)	1474 (18.95)	9511 (17.06)		2760 (18.52)	1380 (18.52)	1380 (18.52)	
2017	12 424 (19.56)	1730 (22.25)	10 694 (19.18)		3222 (21.62)	1611 (21.62)	1611 (21.62)	
Age, y								
Mean (SD)	54.98 (11.92)	64.60 (9.45)	53.63 (11.61)	1.04	65.06 (10.15)	64.33 (9.40)	65.78 (10.81)	0.13
Median (IQR)	54.00 (17.00)	64.00 (13.00)	53.00 (15.00)		64.00 (14.00)	64.00 (12.00)	65.00 (15.00)	
Monthly income, NTD								
Dependent	18 048 (28.41)	3588 (46.14)	14 460 (25.94)	0.47	6933 (46.52)	3385 (45.43)	3548 (47.62)	0.09
<20 000	10 128 (15.94)	1273 (16.37)	8855 (15.88)		2447 (16.42)	1228 (16.48)	1219 (16.36)	
20 000-29 999	18 261 (28.74)	1810 (23.27)	16 451 (29.51)		3444 (23.11)	1748 (23.46)	1696 (22.76)	
≥30 000	17 093 (26.91)	1106 (14.22)	15 987 (28.67)		2078 (13.94)	1090 (14.63)	988 (13.26)	
Stage								
0	3885 (6.12)	448 (5.76)	3437 (6.16)	0.10	835 (5.60)	419 (5.62)	416 (5.58)	0.02
1	22 194 (34.93)	2868 (36.88)	19 326 (34.66)		5553 (37.26)	2751 (36.92)	2802 (37.61)	
2	27 228 (42.86)	3405 (43.78)	23 823 (42.73)		6460 (43.35)	3256 (43.70)	3204 (43.00)	
3	5830 (9.18)	609 (7.83)	5221 (9.36)		1200 (8.05)	587 (7.88)	613 (8.23)	
4	4393 (6.91)	447 (5.75)	3946 (7.08)		854 (5.73)	438 (5.88)	416 (5.58)	
Therapies								
Radiotherapy	1934 (3.04)	276 (3.55)	1658 (2.97)	0.03	527 (3.54)	262 (3.52)	265 (3.56)	0.002
Operation								
Lumpectomy	20 195 (31.79)	2691 (34.60)	17 504 (31.40)	0.08	5125 (34.39)	2511 (33.70)	2614 (35.08)	0.02
Mastectomy	19 320 (30.41)	2463 (31.67)	16 857 (30.24)		4705 (31.57)	2401 (32.22)	2304 (30.92)	
No surgery	24 015 (37.80)	2623 (33.73)	21 392 (38.37)		5072 (34.04)	2539 (34.08)	2533 (34.00)	
Adjuvant therapy	28 615 (45.04)	4000 (51.43)	24 615 (44.15)	0.15	7629 (51.19)	3808 (51.11)	3821 (51.28)	0.06
Neoadjuvant therapy	8727 (13.74)	827 (10.63)	7900 (14.17)		1542 (10.35)	790 (10.60)	752 (10.09)	
Hormone therapy								
Tamoxifen	13 903 (21.88)	1317 (16.93)	12 586 (22.57)	0.14	2534 (17.00)	1288 (17.29)	1246 (16.72)	0.02
Aromatase inhibitors	9444 (14.87)	2135 (27.45)	7309 (13.11)	0.36	4153 (27.87)	1991 (26.72)	2162 (29.02)	0.05
Trastuzumab	3765 (5.93)	410 (5.27)	3355 (6.02)	0.03	764 (5.13)	396 (5.31)	368 (4.94)	0.02
Anthracyclines	27 327 (43.01)	2914 (37.47)	24 413 (43.79)	0.13	5511 (36.98)	2808 (37.69)	2703 (36.28)	0.03
Taxanes	9158 (14.42)	868 (11.16)	8290 (14.87)	0.11	1637 (10.99)	838 (11.25)	799 (10.72)	0.02
5-fluorouracil	17 720 (27.89)	2035 (26.17)	15 685 (28.13)	0.04	3823 (25.65)	1965 (26.37)	1858 (24.94)	0.03
Cyclophosphamide	31 615 (49.76)	3469 (44.61)	28 146 (50.48)	0.12	6536 (43.86)	3342 (44.85)	3194 (42.87)	0.04
CV medications								
ACEI/ARB	9468 (14.90)	3670 (47.19)	5798 (10.40)	0.89	6759 (45.36)	3407 (45.73)	3352 (44.99)	0.01
β-blocker	9058 (14.26)	2570 (33.05)	6488 (11.64)	0.53	4788 (32.13)	2401 (32.22)	2387 (32.04)	0.004
Antiplatelet agents	3363 (5.29)	1634 (21.01)	1729 (3.10)	0.57	2659 (17.84)	1488 (19.97)	1171 (15.72)	0.11
Anticoagulants	366 (0.58)	102 (1.31)	264 (0.47)	0.09	199 (1.34)	96 (1.29)	103 (1.38)	0.008
Digoxin	154 (0.24)	46 (0.59)	108 (0.19)	0.06	90 (0.60)	44 (0.59)	46 (0.62)	0.004
MRA	533 (0.84)	123 (1.58)	410 (0.74)	0.08	229 (1.54)	117 (1.57)	112 (1.50)	0.01
Comorbidities								
CAD	2944 (4.63)	1291 (16.60)	1653 (2.96)	0.47	2259 (15.16)	1181 (15.85)	1078 (14.47)	0.04
PAD	530 (0.83)	171 (2.20)	359 (0.64)	0.13	342 (2.29)	164 (2.20)	178 (2.39)	0.01
Hypertension	15 559 (24.49)	5161 (66.36)	10 398 (18.65)	1.10	10 104 (67.80)	4858 (65.20)	5246 (70.41)	0.11
Diabetes	7824 (12.32)	3942 (50.69)	3882 (6.96)	1.10	6812 (45.71)	3629 (48.70)	3183 (42.72)	0.12
Valve disease	1068 (1.68)	251 (3.23)	817 (1.47)	0.12	511 (3.43)	240 (3.22)	271 (3.64)	0.02
COPD	1084 (1.71)	229 (2.94)	855 (1.53)	0.10	434 (2.91)	214 (2.87)	220 (2.95)	0.005
Asthma	1493 (2.35)	310 (3.99)	1183 (2.12)	0.11	587 (3.94)	295 (3.96)	292 (3.92)	0.002
Atrial fibrillation	279 (0.44)	81 (1.04)	198 (0.36)	0.08	167 (1.12)	78 (1.05)	89 (1.19)	0.01
CKD or ESKD	1671 (14.51)	666 (8.56)	1005 (1.80)	0.31	1187 (7.97)	612 (8.21)	575 (7.72)	0.02

Abbreviations: ACEI/ARB, angiotensin-converting enzyme inhibitor/angiotensin receptor blocker; ASMD, absolute standardized mean difference; CAD, coronary artery disease; CKD, chronic kidney disease; COPD, chronic obstructive pulmonary disease; CV, cardiovascular; ESKD, end-stage kidney disease; MRA, mineralocorticoid-receptor antagonists; PAD, peripheral artery disease.

### Risks of Death and Cardiovascular Outcomes Between Patients With Breast Cancer Receiving Statins and Those Not Receiving Statins

**Figure 1.**
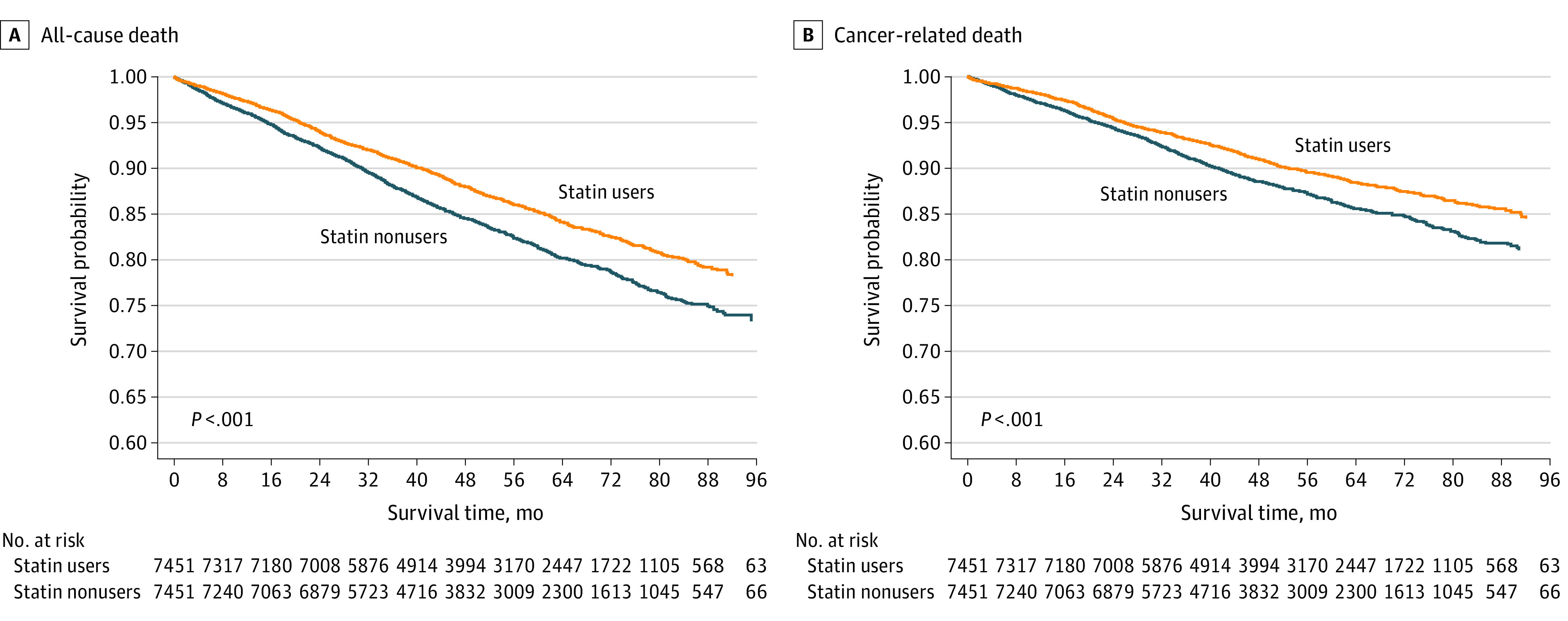
Estimated Probabilities of All-Cause and Cancer-Associated Death Among Patients With Breast Cancer Receiving or Not Receiving Statins

Compared with statin nonusers, statin users had a lower risk of all-cause death (crude HR, 0.78; 95% CI, 0.72-0.85; *P* < .001) (Table 2). After adjusting for imbalanced parameters (ie, ASMD >0.1 in Table 1), including age, antiplatelet agent use, hypertension, and diabetes, the risk of all-cause death remained lower among statin users than among nonusers (adjusted HR, 0.83; 95% CI, 0.77-0.91; *P* < .001). To further investigate the reasons for death, we identified patients with cancer death, cardiovascular death, or other cause of death. Notably, only a small number of patients died due to cardiovascular reasons, and the ratios were similar between statin users and nonusers (eTable 2 in Supplement 1). In contrast, the risk of cancer death was significantly lower among statin users compared with nonusers (adjusted HR, 0.83; 95% CI, 0.75-0.92; *P* < .001). For instance, by focusing on the 1035 deaths among statin users, we found that there were 56 (5.41%) cardiovascular causes, 739 (71.40%) cancer-associated causes, and 240 (23.19%) other causes. In addition, considering that death may reduce the incidence of cardiovascular events, we analyzed the subdistribution of HRs. Adjusted with other cause of death as a competing risk, cancer-related death was still lower in statin users compared with nonstatin users (subdistribution HR, 0.84; 95% CI, 0.76-0.93; *P* = .001) (Table 3). Considering time-varying exposure to statins, as shown in eTable 3 in Supplement 1, time-dependent analysis of statin exposure showed an even more significant reduction of all-cause (adjusted HR, 0.32; 95% CI, 0.28-0.36; *P* < .001) and cancer-related death (adjusted HR, 0.28; 95% CI, 0.24-0.32; *P* < .001) in statin users compared with nonusers. In the follow-up period up to 8 years, the rate free from death was lower among statin users than among nonusers (Figure 1A). Regarding cancer-associated death, the survival probability was also lower among statin users than among nonusers (Figure 1B). Likewise, the cumulative incidence of cancer death was also higher in non–statin users (eFigure 2 in Supplement 1).

**Table 2.** Crude and Adjusted HRs of Statin Users vs Nonusers Among Patients With Breast Cancer

Outcome	Matched sample (n = 14 902)	Statin users (n = 7451)	Statin nonusers (reference group) (n = 7451)	Crude HR (95% CI)	*P *value	Adjusted HR (95% CI)^a^	*P *value
Primary outcome							
All-cause death	2329 (15.63)	1035 (13.89)	1294 (17.37)	0.78 (0.72-0.85)	<.001	0.83 (0.77-0.91)	<.001
Cardiovascular death	126 (0.85)	56 (0.75)	70 (0.94)	0.78 (0.55-1.10)	.16	1.01 (0.70-1.45)	.97
Cancer death	1652 (11.09)	739 (9.92)	913 (12.25)	0.79 (0.72-0.87)	<.001	0.83 (0.75-0.92)	<.001
Secondary outcome							
Heart failure	1093 (7.33)	524 (7.03)	569 (7.64)	0.90 (0.80-1.01)	.08	0.97 (0.86-1.09)	.58
Arterial events^b^	1138 (7.64)	584 (7.84)	554 (7.44)	1.03 (0.92-1.16)	.62	1.07 (0.95-1.20)	.30
Venous events^c^	241 (1.62)	126 (1.69)	115 (1.54)	1.07 (0.83-1.38)	.58	1.12 (0.86-1.44)	.40

Abbreviation: HR, hazard ratio.

^a^ Model was adjusted for age, antiplatelet agents, hypertension, and diabetes (parameters with an absolute standardized mean difference greater than 0.1 in Table 1).

^b^ Arterial events included acute myocardial infarction and ischemic strokes.

^c^ Venous events included pulmonary embolism and deep venous thrombosis.

**Table 3.** Crude and Adjusted sHRs of Statin Users and Nonusers Among Patients With Breast Cancer

Outcome	Matched sample (n = 14 902)	Statin user (n = 7451)	Statin nonusers (reference group) (n = 7451)	Crude sHR (95% CI)	*P *value	Adjusted sHR (95% CI)^a^	*P *value
Primary outcome							
Cardiovascular death	126 (0.85)	56 (0.75)	70 (0.94)	0.80 (0.56-1.13)	.21	1.06 (0.74-1.52)	.74
Cancer death	1652 (11.09)	739 (9.92)	913 (12.25)	0.80 (0.72-0.88)	<.001	0.84 (0.76-0.93)	.001
Secondary outcome							
Heart failure	1093 (7.33)	524 (7.03)	569 (7.64)	0.92 (0.82-1.03)	.16	0.99 (0.88-1.12)	.93
Arterial events^b^	1138 (7.64)	584 (7.84)	554 (7.44)	1.05 (0.94-1.18)	.39	1.09 (0.97-1.23)	.15
Venous events^c^	241 (1.62)	126 (1.69)	115 (1.54)	1.10 (0.85-1.41)	.48	1.14 (0.89-1.45)	.31

Abbreviation: sHR, subdistribution hazard ratio.

^a^ Model was adjusted for age, antiplatelet agents, hypertension, and diabetes (parameters with absolute standardized mean differences >0.1 in Table 1).

^b^ Arterial events included acute myocardial infarction and ischemic strokes.

^c^ Venous events included pulmonary embolism and deep venous thrombosis.

Regarding secondary outcomes focusing on cardiovascular events, the risks of HF and venous events, including pulmonary embolism and DVT, were similar between statin users and nonusers. Despite a numerically lower risk of HF among statin users compared with nonusers (crude HR, 0.90; 95% CI, 0.80-1.01; *P* = .08), the difference was not significant after adjustment (adjusted HR, 0.97; 95% CI, 0.86-1.09; *P* = .58) (Table 2). After adjusting for all-cause death as a competing risk, the risk of secondary outcomes were similar between statin users and nonusers (Table 3). In the time-dependent analysis, the risks of secondary outcomes were not significantly different between the 2 groups (eTable 3 in Supplement 1).

### Subgroup Analysis of Death Between Statin Users and Nonusers

**Figure 2.**
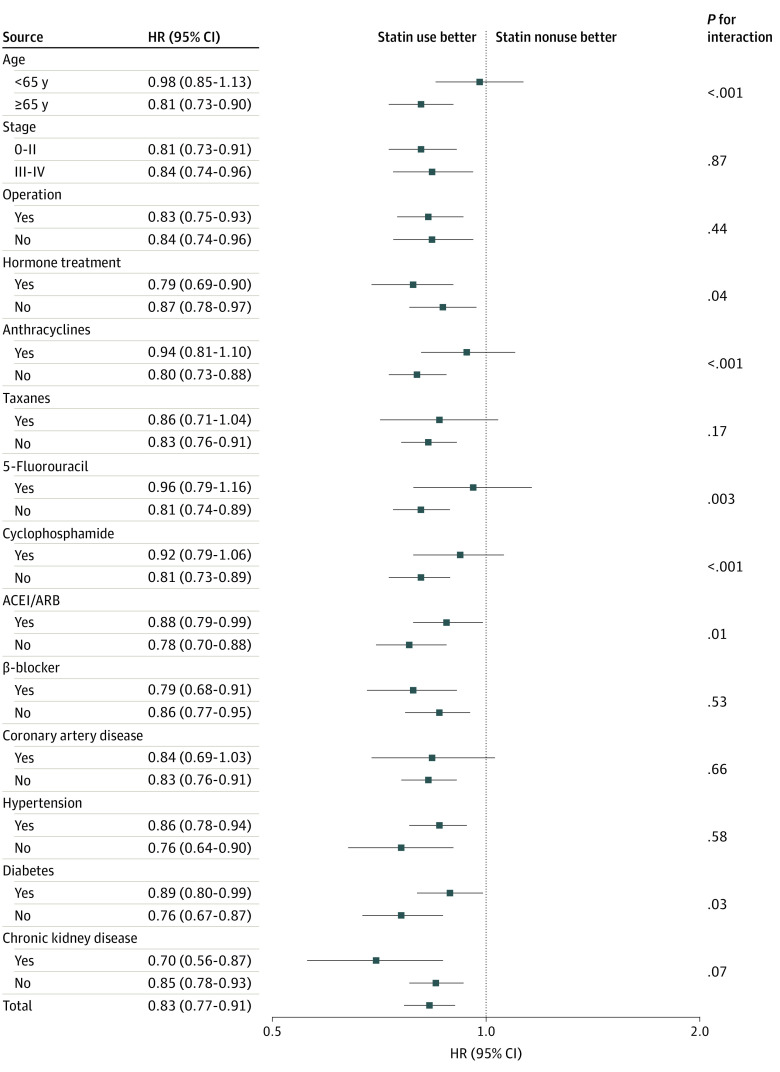
Subgroup Analysis of Risks of All-Cause Death in Patients With Breast Cancer Receiving or Not Receiving Statins ACEI/ARB indicates angiotensin-converting enzyme inhibitor/angiotensin receptor blocker; HR, hazard ratio.

According to our findings, statin use was associated with a reduced risk of death in patients with breast cancer, and whether this phenomenon could be observed in populations with different characteristics remains uncertain. In the subgroup analysis, we found that the reduced risk of death among statin users was independent of cancer stage, operations, antihormone therapies, cardiovascular drugs, history of hypertension, diabetes, or chronic kidney disease (Figure 2). Among patients who were younger than 65 years and those who received anticancer drugs, including anthracyclines, taxanes, 5-fluorouracil, and cyclophosphamide, the benefit of statin use was not significant. The benefit of statin use diminished among patients with a history of CAD.

### Association of HDS Use With Mortality and Cardiovascular Outcomes

To investigate whether different types and doses of statins were associated with the outcomes, we compared the mortality and cardiovascular outcomes among HDS users, non-HDS users, and statin nonusers. The details of HDS and non-HDS statin users are shown in eTable 4 in Supplement 1. After propensity score matching, a total of 9672 patients with breast cancer were included with 1:1:1 matching (HDS users vs non-HDS users vs statin nonusers) (eTable 5 in Supplement 1). The causes of death among the 3 groups are displayed in eTable 6 in Supplement 1. Compared with statin nonusers, the risk of all-cause mortality was reduced among non-HDS users (adjusted HR, 0.87; 95% CI, 0.77-0.99; *P* = .03) but was even lower in HDS users (adjusted HR, 0.82; 95% CI, 0.72-0.93; *P* = .002). The risk of cancer-related death was persistently low in both HDS (adjusted HR, 0.78; 95% CI, 0.67-0.90; *P* = .001) and non-HDS (adjusted HR, 0.84; 95% CI, 0.73-0.97; *P* = .002) users compared with statin nonusers (eTable 7 in Supplement 1). In contrast, the risks of cardiovascular death and other cardiovascular outcomes were similar among the 3 groups. Setting other cause of death as a competing risk, we observed a consistent result. Compared with statin nonusers, the risk of cancer-related death was reduced in non-HDS users (adjusted HR, 0.84; 95% CI, 0.73-0.98; *P* = .002) and was even lower in HDS users (adjusted HR, 0.79; 95% CI, 0.68-0.91; *P* = 001), while there were no significant differences in terms of the risks of cardiovascular death and other cardiovascular outcomes (eTable 8 in Supplement 1).

Taken together, despite statins being drugs for lowering lipids and protecting the cardiovascular system, we found a significant risk reduction of death, especially cancer-associated death, among patients with breast cancer who received statins, especially HDS. Surprisingly, no significant differences were observed in the cardiovascular outcomes between statin users and nonusers.

## Discussion

In this nationwide cohort, we found that among patients with breast cancer, the use of statins was associated with a significant risk reduction of cancer-related deaths. In contrast, cardiovascular outcomes were not significantly different between statin users and nonusers. Statins are known to suppress atherosclerosis and improve cardiovascular outcomes.^4,14,15^ In a retrospective cohort study, Abdel-Qadir et al^16^ reported that statin exposure lowered the risk of hospitalization for HF in women with breast cancer aged 66 years or older. It has been speculated that patients prescribed statins have a high prevalence of preexisting coronary heart disease and face excess cardiac risks. However, in our cohort focusing on Asian patients with breast cancer, who were relatively younger at diagnosis, only 5% to 7% of the patients had underlying vascular diseases. On the other hand, statins have received special attention for their pleiotropic effects, including anti-inflammatory and antitumor potential.^4,5,6^ A large number of clinical and epidemiological studies have described the anticancer properties of statins, but the evidence for the anticancer effects of statins is inconsistent.^4,5,6,17^ In a cohort of 3165 women with breast cancer,^17^ statin use was consistently associated with reduced mortality. In a retrospective analysis of 748 patients with non–small cell lung cancer, Atkins et al^18^ reported statin use as an independent predictor of all-cause death. In contrast, through analyzing data from Surveillance, Epidemiology, and End Results from 2007 to 2009, Emilsson et al^19^ observed that among 17 372 patients with various types cancers, statin exposure within 6 months after cancer diagnosis failed to improve 3-year survival. Our study focused on the outcomes of pretreatment with statins (within 6 months before diagnosis of breast cancer) and found a significantly better cancer-specific and overall survival among statin users compared with nonusers. The benefit of statins in preventing cardiovascular morbidities may not be as significant as in other studies, but their potential association with reducing cancer-associated death was highlighted.

Mechanistically, it remains unclear how statins suppress the growth and metastasis of cancer cells. A previous study reported that by blocking the mevalonate pathway, statins effectively disrupted estrogen receptor expression, cancer cell survival, and proliferation.^15^ Similarly, in an in vitro study, Choe et al^14^ found that atorvastatin reduced programmed cell death ligand 1 (PD-L1) expression in breast cancer cells, thus enhancing the efficacy of anti–PD-L1 therapy. In addition, using a xenograft model of metastatic triple-negative breast cancer, Marti et al^3^ found that, in addition to doxorubicin, statins expressed a synergic effect to reduce cancer progression. Based on the association of statins with blocking metastatic tumor outgrowth in both 2-dimensional in vitro and 3-dimensional ex vivo models, Beckwitt et al^15^ suggested the long-term use of statins as adjuvant drugs to decrease death in patients with breast cancer. In this Asian cohort, we also found a significant risk reduction of mortality, especially cancer-associated death, among patients with breast cancer who received statins. Notably, in the subgroup analysis, we found that in conjunction with anticancer drugs, statins also exhibited a trend toward better survival. Given that patients with breast cancer in Asia are relatively younger at diagnosis and most are free from traditional cardiovascular risk factors,^8,9,20^ statin use is less common among this specific population. In this population-based investigation, statin use was associated with a reduced risk of cancer-associated mortality rather than cardiovascular death. Thus, our findings provide evidence supporting the potential benefits of statin use in patients with breast cancer.

### Limitations

Our study has some limitations. First, given that statin users may have a higher risk of cardiovascular disease than nonusers, selection bias may exist. To avoid the influence of existing cardiovascular risks, we carefully matched age, stage, comorbidities, cardiovascular disease, and anticancer drugs. Also, using a time-dependent analysis, we set statin exposure time as a segmented time-dependent covariate and observed a consistent result of a reducing risk of cancer-related death in statin users compared with nonusers. Also, in the design of an administrative claims database, clinical information, including blood test results (eg, low-density lipoprotein); estrogen, progesterone, and *EBBR2* expression; menopause status; and social and family histories are missing.

## Conclusions

Using this nationwide Asian cohort, we observed a significantly lower risk of cancer-related death, but not cardiovascular death, among patients with breast cancer receiving statins than among those who did not receive statins. Other cardiovascular outcomes, including HF and arterial and venous events, were similar between statin users and nonusers. More investigations, especially randomized clinical trials, are necessary to support our findings.

## Notes

Supplement 1. **eTable 1.** *ICD-9* and *ICD-10* Codes **eTable 2. **Causes of Death Between Statin Users and Nonusers **eTable 3**. Crude and Adjusted Hazard Ratio (HR) of Statin Users and Nonusers Among Patients With Breast Cancer Using Time-Dependent Method **eTable 4. **Initial Regimens of High-Dose Statins (HDS) and Non-HDS **eTable 5. **Baseline Characteristics of High-Dose Statin (HDS) Users, non-HDS Users, and Statin Nonusers Among Patients With Breast Cancer After Propensity Score Matching **eTable 6. **Causes of Death Among High-Dose Statin (HDS) Users, Non-HDS Users, and Statin Nonusers Among Patients With Breast Cancer **eTable 7. **Crude and Adjusted Hazard Ratio (HR) of High-Dose Statin (HDS) Users, Non-HDS Users, and Statin Nonusers Among Patients With Breast Cancer **eTable 8. **Crude and Adjusted Subdistribution Hazard Ratio (sHR) of High-Dose Statin (HDS) Users, Non-HDS Users, and Statin Nonusers Among Patients With Breast Cancer **eFigure 1.** Flowchart of Study Design **eFigure 2.** Results of the Cumulative Incidence of Cancer Death Adjusted With Competing Risk Events of Other Reason Death

Supplement 2.
Data Sharing Statement
