# Supplementary material for: Association of Statin Use With Cancer- and Noncancer-Associated Survival Among Patients With Breast Cancer in Asia
Source: JAMA Netw Open. 2023 Apr 21;6(4):e239515. doi: 10.1001/jamanetworkopen.2023.9515 (PMC10122177; doi:10.1001/jamanetworkopen.2023.9515)
Supplement: Supplement 1. — eTable 1. ICD-9 and ICD-10 Codes eTable 2. Causes of Death Between Statin Users and Nonusers eTable 3. Crude and Adjusted Hazard Ratio (HR) of Statin Users and Nonusers Among Patients With Breast Cancer Using Time-Dependent Method eTable 4. Initial Regimens of High-Dose Statins (HDS) and Non-HDS eTable 5. Baseline Characteristics of High-Dose Statin (HDS) Users, non-HDS Users, and Statin Nonusers Among Patients With Breast Cancer After Propensity Score Matching eTable 6. Causes of Death Among High-Dose Statin (HDS) Users, Non-HDS Users, and Statin Nonusers Among Patients With Breast Cancer eTable 7. Crude and Adjusted Hazard Ratio (HR) of High-Dose Statin (HDS) Users, Non-HDS Users, and Statin Nonusers Among Patients With Breast Cancer eTable 8. Crude and Adjusted Subdistribution Hazard Ratio (sHR) of High-Dose Statin (HDS) Users, Non-HDS Users, and Statin Nonusers Among Patients With Breast Cancer eFigure 1. Flowchart of Study Design eFigure 2. Results of the Cumulative Incidence of Cancer Death Adjusted With Competing Risk Events of Other Reason Death [file jamanetwopen-e239515-s001.pdf]

Supplemental Online Content

Chang WT, Lin HW, Lin SH, Li YH. Association of statin use with cancer- and noncancer-associated survival among patients with breast cancer in Asia. *JAMA Netw Open.* 2023;6(4):e239515. doi:10.1001/jamanetworkopen.2023.9515

eTable 1. *ICD-9* and *ICD-10* Codes

eTable 2. Causes of Death Between Statin Users and Nonusers

eTable 3 . Crude and Adjusted Hazard Ratio (HR) of Statin Users and Nonusers Among Patients With Breast Cancer Using Time-Dependent Method

eTable 4. Initial Regimens of High-Dose Statins (HDS) and Non-HDS

eTable 5. Baseline Characteristics of High-Dose Statin (HDS) Users, non-HDS Users, and Statin Nonusers Among Patients With Breast Cancer After Propensity Score Matching

eTable 6. Causes of Death Among High-Dose Statin (HDS) Users, Non-HDS Users, and Statin Nonusers Among Patients With Breast Cancer

eTable 7. Crude and Adjusted Hazard Ratio (HR) of High-Dose Statin (HDS) Users, Non-HDS Users, and Statin Nonusers Among Patients With Breast Cancer

eTable 8. Crude and Adjusted Subdistribution Hazard Ratio (sHR) of High-Dose Statin (HDS) Users, Non-HDS Users, and Statin Nonusers Among Patients With Breast Cancer

eFigure 1. Flowchart of Study Design

eFigure 2. Results of the Cumulative Incidence of Cancer Death Adjusted With Competing Risk Events of Other Reason Death

This supplemental material has been provided by the authors to give readers additional information about their work.

© 2023 Chang WT et al. *JAMA Network Open.*

eTable 1. *ICD-9* and *ICD-10* Codes

Disease	ICD-9 Codes	ICD-10 Codes
Breast cancer	174	C50
Outcome		
Acute myocardial infarction	410	I21, I22, I23
Heart failure	428, 402.01, 402.11, 402.91, 404.01,<br>404.03, 404.11, 404.13, 404.91, 404.93, 785.51	I11.0, I13.0, I13.2, I42.0, I42.1, I42.2, I42.3, I42.4, I42.5, I42.6, I42.7, I42.8, I42.9, I43, I50.1, I50.20, I50.21, I50.22, I50.23, I50.30, I50.31, I50.32, I50.33, I50.40, I50.41, I50.42, I50.43, I50.9, R57.0
Ischemic stroke (including TIA)	433, 434, 435, 436	I63, I65, I66, I67.89, I67.84, G45.0, G45.1, G45.2, G45.8, G45.9, G46.0, G46.1, G46.2
Pulmonary embolism	415.1	I26, I26.02, I27.82, I26.92 I26.93, I26.99
Deep venous thrombosis	451.11, 453.8, 453.4,453.40, 453.9, 451.83	I82
Comorbidities		
Coronary artery disease	410, 411, 412, 413, 414	I20, I21, I22, I24, I25
Peripheral artery disease	440, 443, 444, 447.8, 447.9	I70.2-I70.9, I71, I73.9, I74.2, I74.3, I74.4, I74.5, I77.89, I77.9
Hypertension	401, 402, 403, 404, 405	I10, I11.0, I11.9, I12.0, I12.9, I13.0, I13.2, I13.11, I15, N26.2
Diabetes mellitus	250	E08, E09, E11, E13
Valve disorders	394-397, 424.0, 424.1, 424.2, 424.3	I05, I06, I07, I08, I09, I34-I37
Chronic obstructive pulmonary disease	491, 492, 494, 495, 496	J41, J42, J43, J44, J47, J67
Asthma	493	J45
Atrial fibrillation	427.31, 427.32	I48
Chronic kidney disease	580-589, 403, 404, 585, V45.1, V56	I12, I13, N02, N03, N04, N05, N06, N07, N08, N11, N14, N17, N18, N19, N29, O10.2, O10.3, Q61, Z49, Z99.2
ESRD	585	N18.6; Z99.2
Procedure Code		
Lumpectomy	85.21	0HTT, 0HTU, 0HTV, 0HBT, 0HBX
Mastectomy	85.4	0H5T, 0H5U, 0H5V, 0H5X, 0H5Y, 0HPT, 0HPU
Radiotherapy	V58.0	Z51.0

© 2023 Chang WT et al. *JAMA Network Open.*

eTable 2. Causes of Death Between Statin Users and Nonusers

	Total N=2329		Statin users N=1035		Statin non-users N=1294	
Cardiovascular death	126	(5.41)	56	(5.41)	70	(5.41)
Cancer associated death	1652	(70.93)	739	(71.40)	913	(70.56)
Other reasons	551	(23.66)	240	(23.19)	311	(24.03)

© 2023 Chang WT et al. *JAMA Network Open.*

eTable 3. Crude and Adjusted Hazard Ratio (HR) of Statin Users and Nonusers Among Patients With Breast Cancer Using Time-Dependent Method

	Total N=14902	Statin users N=7451	Statin non-users (Ref) N=7451	Crude HR (95%CI)	*p* value	Adjusted HR (95%CI)	*p* value
**Primary outcome**							
All cause death	2329 (15.63)	1035 (13.89)	1294 (17.37)	0.30 (0.27-0.34)	<0.001	0.32 (0.28-0.36)	<0.001
Cardiovascular death	126 (0.85)	56 (0.75)	70 (0.94)	0.55 (0.36-0.82)	0.005	0.61 (0.40-0.92)	0.02
Cancer death	1652 (11.09)	739 (9.92)	913 (12.25)	0.26 (0.23-0.30)	<0.001	0.28 (0.24-0.32)	<0.001
**Secondary outcome**							
Heart failure	1093 (7.33)	524 (7.03)	569 (7.64)	0.94 (0.83-1.07)	0.37	0.96 (0.84-1.09)	0.50
Arterial events[a]	1138 (7.64)	584 (7.84)	554 (7.44)	1.00 (0.88-1.13)	0.95	1.01 (0.89-1.14)	0.94
Venous events[b]	241 (1.62)	126 (1.69)	115 (1.54)	0.88 (0.66-1.16)	0.39	0.90 (0.67-1.19)	0.45

Model was adjusted for age, anti-platelet agents, hypertension, diabetes mellitus, and statin exposure time as time-dependent covariate (parameters with ASMD > 0.1 in Table 1).

[a]Arterial events including acute myocardial infarction ad ischemic strokes.

[b]Venous events including pulmonary embolism and deep venous thrombosis.

© 2023 Chang WT et al. *JAMA Network Open.*

eTable 4. Initial Regimens of High-Dose Statins (HDS) and Non-HDS

	Non-HDS users					HDS users[a]			
	Atorvastatin 10 mg	Rosuvastatin 5 mg	Simvastatin 10 mg	Simvastatin 20 mg	Others	Atorvastatin 20 mg	Atorvastatin 40 mg	Rosuvastatin 10 mg	Simvastatin 40 mg
N	1941	238	47	735	1592	944	286	1915	79
%	24.96%	3.06%	0.60%	9.45%	20.47%	12.14%	3.68%	24.62%	1.02%

[a]HDS were defined as ≥ 10 mg rosuvastatin, ≥ 20 mg atorvastatin, and ≥ 40 mg simvastatin at the initial regimens.

© 2023 Chang WT et al. *JAMA Network Open.*

eTable 5. Baseline Characteristics of High-Dose Statin (HDS) Users, non-HDS Users, and Statin Nonusers Among Patients With Breast Cancer After Propensity Score Matching

	Total N=9672		HDS users N=3224		Non-HDS users N=3224		Statin non-users N=3224		ASMD		
									a vs c	b vs c	a vs b
Year									0.00	0.00	0.00
2012	1215	(12.56)	405	(12.56)	405	(12.56)	405	(12.56)			
2013	1329	(13.74)	443	(13.74)	443	(13.74)	443	(13.74)			
2014	1569	(16.22)	523	(16.22)	523	(16.22)	523	(16.22)			
2015	1746	(18.05)	582	(18.05)	582	(18.05)	582	(18.05)			
2016	1728	(17.87)	576	(17.87)	576	(17.87)	576	(17.87)			
2017	2085	(21.56)	695	(21.56)	695	(21.56)	695	(21.56)			
Age									0.12	0.11	0.001
Mean (SD)	65.08	(9.92)	64.68	(9.30)	64.69	(9.62)	65.85	(10.76)			
Median (IQR)	64.00	(14.00)	65.00	(13.00)	64.00	(13.00)	65.00	(16.00)			
Monthly income, NTD									0.06	0.05	0.07
Dependent	4499	(46.52)	1480	(45.91)	1483	(46.00)	1536	(47.64)			
<20,000	1576	(16.29)	527	(16.35)	530	(16.44)	519	(16.10)			
20,000-29,999	2217	(22.92)	717	(22.24)	786	(24.38)	714	(22.15)			
≧30,000	1380	(14.27)	500	(15.51)	425	(13.18)	455	(14.11)			
Stage									0.06	0.08	0.06
0	564	(5.83)	187	(5.80)	185	(5.74)	192	(5.96)			
1	3651	(37.75)	1218	(37.78)	1190	(36.91)	1243	(38.55)			
2	4211	(43.54)	1427	(44.26)	1382	(42.87)	1402	(43.49)			
3	680	(7.03)	221	(6.85)	258	(8.00)	201	(6.23)			

© 2023 Chang WT et al. *JAMA Network Open.*

4	566	(5.85)	171	(5.30)	209	(6.48)	186	(5.77)			
Therapies use during											
Radiotherapy	328	(3.39)	104	(3.23)	115	(3.57)	109	(3.38)	0.009	0.01	0.02
Operation									0.04	0.04	0.04
Lumpectomy	3274	(33.85)	1094	(33.93)	1062	(32.94)	1118	(34.68)			
Mastectomy	3116	(32.22)	1067	(33.10)	1034	(32.07)	1015	(31.48)			
No surgery	3282	(33.93)	1063	(32.97)	1128	(34.99)	1091	(33.84)			
Adjuvent therapy	4982	(51.51)	1708	(52.98)	1604	(49.75)	1670	(51.80)	0.02	0.05	0.06
Neoadjuvent therapy	989	(10.23)	315	(9.77)	359	(11.14)	315	(9.77)			
Hormone Tx											
Tamoxifen	1713	(17.71)	595	(18.46)	517	(16.04)	601	(18.64)	0.005	0.07	0.06
Aromatase inhibitors	2667	(27.57)	864	(26.80)	895	(27.76)	908	(28.16)	0.03	0.009	0.02
Trastuzumab	516	(5.33)	156	(4.84)	179	(5.55)	181	(5.61)	0.03	0.003	0.03
Anthracyclines	3548	(36.68)	1179	(36.57)	1218	(37.78)	1151	(35.70)	0.02	0.04	0.03
Taxanes	1014	(10.48)	337	(10.45)	351	(10.89)	326	(10.11)	0.01	0.03	0.01
5-fluorouracil	2503	(25.88)	823	(25.53)	860	(26.67)	820	(25.43)	0.002	0.03	0.03
Cyclophosphamide	4246	(43.90)	1420	(44.04)	1450	(44.98)	1376	(42.68)	0.03	0.05	0.02
CV medications											
ACEI/ARB	4592	(47.48)	1554	(48.20)	1494	(46.34)	1544	(47.89)	0.006	0.03	0.04
Beta blocker	3205	(33.14)	1082	(33.56)	1037	(32.17)	1086	(33.68)	0.003	0.03	0.03
Anti-platelet agents	1965	(20.32)	735	(22.80)	633	(19.63)	597	(18.52)	0.11	0.03	0.08
Anti-coagulants	128	(1.32)	42	(1.30)	42	(1.30)	44	(1.36)	0.005	0.005	0.00
Digoxin	63	(0.65)	18	(0.56)	18	(0.56)	27	(0.84)	0.03	0.03	0.00
MRA	151	(1.56)	45	(1.40)	58	(1.80)	48	(1.49)	0.008	0.02	0.03
Comorbidities											

© 2023 Chang WT et al. *JAMA Network Open.*

CAD	1597	(16.51)	549	(17.03)	546	(16.94)	502	(15.57)	0.04	0.04	0.003
PAD	226	(2.34)	73	(2.26)	74	(2.30)	79	(2.45)	0.01	0.01	0.002
Hypertension	6576	(67.99)	2123	(65.85)	2170	(67.31)	2283	(70.81)	0.11	0.08	0.03
Diabetes mellitus	4772	(49.34)	1610	(49.94)	1633	(50.65)	1529	(47.43)	0.05	0.06	0.01
Valve disease	329	(3.40)	119	(3.69)	91	(2.82)	119	(3.69)	0.00	0.05	0.05
COPD	284	(2.94)	89	(2.76)	101	(3.13)	94	(2.92)	0.01	0.01	0.02
Asthma	388	(4.01)	127	((3.94)	131	(4.06)	130	(4.03)	0.005	0.002	0.006
Atrial fibrillation	110	(1.14)	36	(1.12)	37	(1.15)	37	(1.15)	0.003	0.00	0.003
CKD/ESRD	824	(8.52)	272	(8.44)	293	(9.09)	259	(8.03)	0.01	0.04	0.02

ASMD= absolute standardized mean difference; CV=cardiovascular; ACEI/ARB= angiotensin-converting enzyme inhibitor/Angiotensin Receptor Blocker; MRA= mineralocorticoid-receptor antagonists; CAD=coronary artery disease; PAD=peripheral artery disease; COPD=chronic obstructive pulmonary disease; CKD= Chronic kidney disease; ESRD=end-stage renal disease

a indicate HDS users

b indicate Non-HDS users

c indicate Statin non-users

© 2023 Chang WT et al. *JAMA Network Open.*

eTable 6. Causes of Death Among High-Dose Statin (HDS) Users, Non-HDS Users, and Statin Nonusers Among Patients With Breast Cancer

	Total N=1472		HDS users N=440		Non-HDS users N=466		Statin non-users N=566	
Cardiovascular death	77	(5.23)	29	(6.59)	23	(4.94)	25	(4.42)
Cancer associated death	1053	(71.54)	310	(70.45)	331	(71.03)	412	(72.79)
Other reasons	342	(23.23)	101	(22.95)	112	(24.03)	129	(22.79)

© 2023 Chang WT et al. *JAMA Network Open.*

eTable 7. Crude and Adjusted Hazard Ratio (HR) of High-Dose Statin (HDS) Users, Non-HDS Users, and Statin Nonusers Among Patients With Breast Cancer

	Total N=9672	HDS users N=3224	Non-HDS users N=3224	Statin non-users (Ref) N=3224		Crude HR (95%CI)	*p* value	Adjusted HR (95%CI)	*p* value
**Primary outcome**									
All cause death	1472 (15.22)	440 (13.65)	466 (14.45)	566 (17.56)	H	0.76 (0.67-0.86)	<0.001	0.82 (0.72-0.93)	0.002
					N	0.81 (0.71-0.91)	0.001	0.87 (0.77-0.99)	0.03
Cardiovascular death	77 (0.80)	29 (0.90)	23 (0.71)	25 (0.78)	H	1.13 (0.66-1.92)	0.67	1.41 (0.82-2.42)	0.22
					N	0.89 (0.51-1.58)	0.70	1.13 (0.64-1.99)	0.68
Cancer death	1053 (10.89)	310 (9.62)	331 (10.27)	412 (12.78)	H	0.73 (0.63-0.85)	<0.001	0.78 (0.67-0.90)	0.001
					N	0.79 (0.68-0.91)	0.001	0.84 (0.73-0.97)	0.02
**Secondary outcome**									
Heart failure	748 (7.73)	253 (7.85)	223 (6.92)	272 (8.44)	H	0.91 (0.77-1.08)	0.29	0.97 (0.82-1.16)	0.75
					N	0.80 (0.67-0.96)	0.01	0.87 (0.73-1.04)	0.13
Arterial events[a]	780 (8.06)	282 (8.75)	247 (7.66)	251 (7.79)	H	1.10 (0.93-1.31)	0.26	1.16 (0.97-1.37)	0.10
					N	0.96 (0.81-1.15)	0.68	1.03 (0.87-1.23)	0.72
Venous events[b]	155 (1.60)	61 (1.89)	51 (1.58)	43 (1.33)	H	1.39 (0.94-2.06)	0.10	1.42 (0.96-2.10)	0.08
					N	1.17 (0.78-1.75)	0.46	1.19 (0.80-1.79)	0.39

Model was adjusted for age, anti-platelet agents, hypertension. (parameters with ASMD >0.1 in Table 1)

H= high dose statin users, N= non-high dose statin users

[a]Arterial events including acute myocardial infarction and ischemic strokes.

[b]Venous events including pulmonary embolism and deep venous thrombosis.

© 2023 Chang WT et al. *JAMA Network Open.*

eTable 8. Crude and Adjusted Subdistribution Hazard Ratio (sHR) of High-Dose Statin (HDS) Users, Non-HDS Users, and Statin Nonusers Among Patients With Breast Cancer

	Total N=9672	HDS users N=3224	Non-HDS users N=3224	Statin non-users (Ref) N=3224		Crude sHR (95%CI)	*p* value	Adjusted sHR (95%CI)	*p* value
**Primary outcome**									
Cardiovascular death	77 (0.80)	29 (0.90)	23 (0.71)	25 (0.78)	H	1.16 (0.68-1.99)	0.58	1.49 (0.86-2.56)	0.16
					N	0.92 (0.52-1.61)	0.76	1.16 (0.66-2.02)	0.61
Cancer death	1053 (10.89)	310 (9.62)	331 (10.27)	412 (12.78)	H	0.74 (0.64-0.85)	<0.001	0.79 (0.68-0.91)	0.001
					N	0.79 (0.69-0.92)	0.002	0.84 (0.73-0.98)	0.02
**Secondary outcome**									
Heart failure	748 (7.73)	253 (7.85)	223 (6.92)	272 (8.44)	H	0.93 (0.79-1.11)	0.42	1.01 (0.85-1.20)	0.95
					N	0.81 (0.68-0.97)	0.02	0.89 (0.74-1.06)	0.19
Arterial events[a]	780 (8.06)	282 (8.75)	247 (7.66)	251 (7.79)	H	1.13 (0.95-1.34)	0.17	1.19 (1.00-1.42)	0.05
					N	0.98 (0.82-1.17)	0.82	1.05 (0.88-1.26)	0.56
Venous events[b]	155 (1.60)	61 (1.89)	51 (1.58)	43 (1.33)	H	1.43 (0.96-2.11)	0.08	1.46 (0.99-2.14)	0.06
					N	1.19 (0.79-1.78)	0.41	1.22 (0.81-1.82)	0.35

Model was adjusted for age, anti-platelet agents, hypertension (parameters with ASMD >0.1 in Table 1).

H= high dose statin users, N= non-high dose statin users

[a]Arterial events including acute myocardial infarction and ischemic strokes.

[b]Venous events including pulmonary embolism and deep venous thrombosis.

© 2023 Chang WT et al. *JAMA Network Open.*

eFigure 1. Flowchart of Study Design

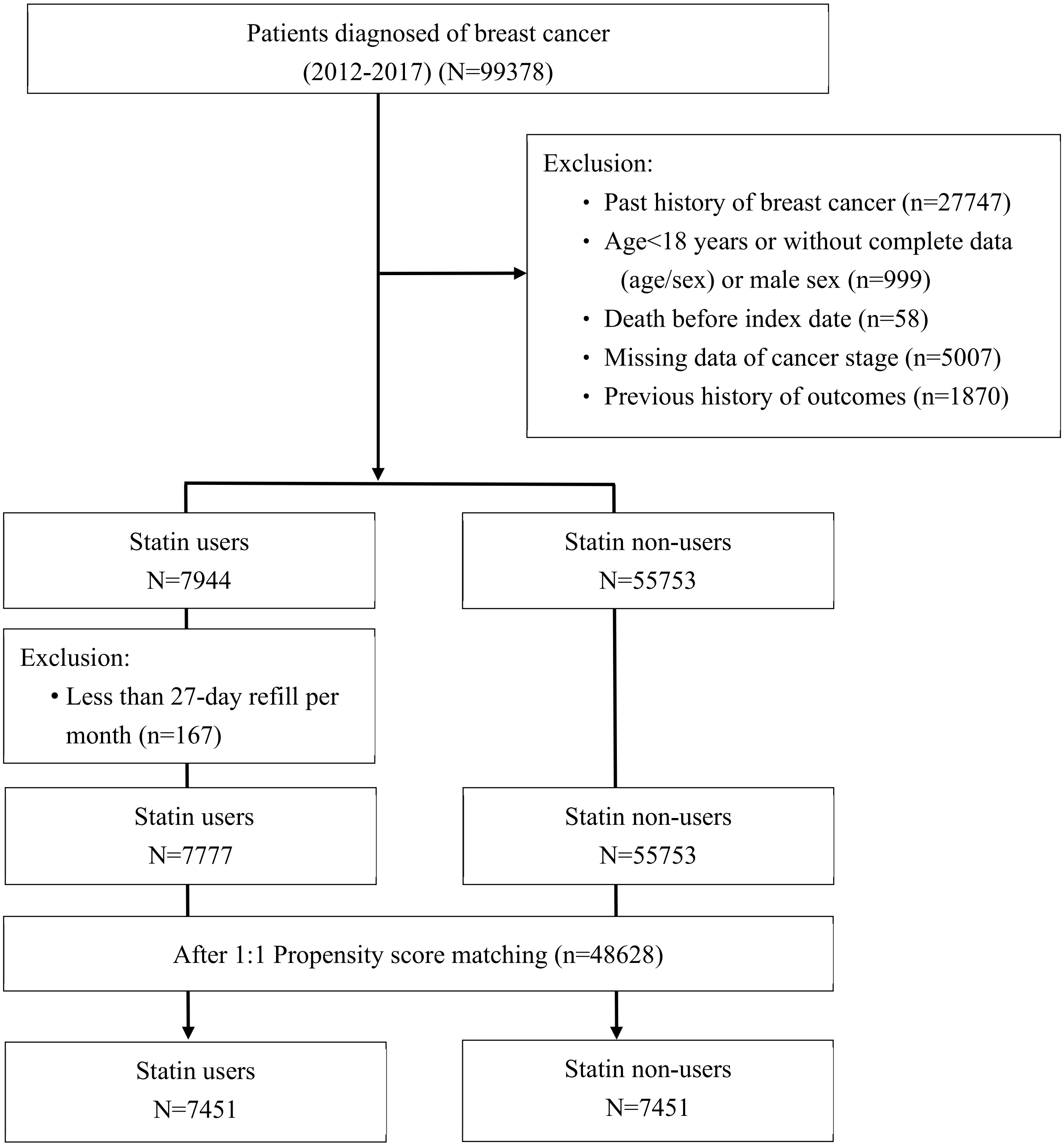

© 2023 Chang WT et al. *JAMA Network Open.*

eFigure 2. Results of the Cumulative Incidence of Cancer Death Adjusted With Competing Risk Events of Other Reason Death

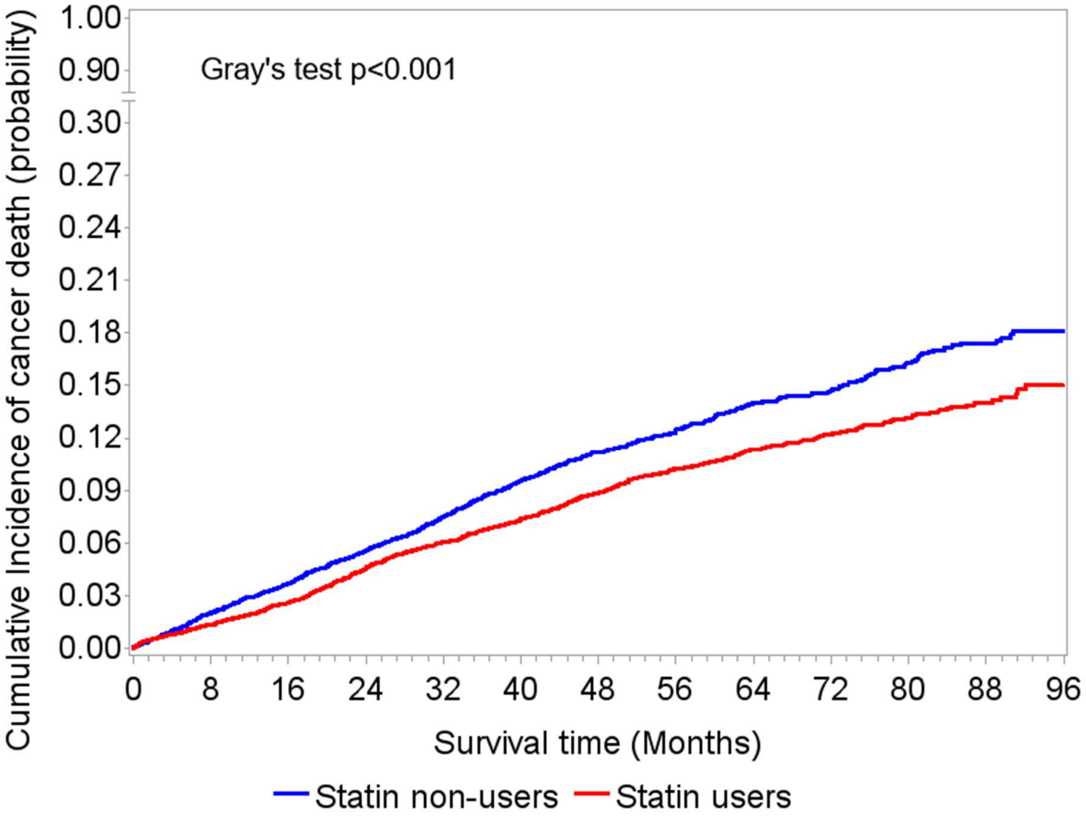

Statin users	7451	7317	7180	7008	5876	4914	3994	3170	2447	1722	1105	568	63
Statin non-users	7451	7240	7063	6879	5723	4716	3832	3009	2300	1613	1045	547	66

© 2023 Chang WT et al. *JAMA Network Open.*
