# Supplementary material for: Association of Statin Use With Cancer- and Noncancer-Associated Survival Among Patients With Breast Cancer in Asia
Source: JAMA Netw Open. 2023 Apr 21;6(4):e239515. doi: 10.1001/jamanetworkopen.2023.9515 (PMC10122177; doi:10.1001/jamanetworkopen.2023.9515)
Supplement: Supplement 2. — Data Sharing Statement [file jamanetwopen-e239515-s002.pdf]

Data Sharing Statement

Chang. Association of Statin Use With Cancer- and Noncancer-Associated Survival Among Patients With Breast Cancer in Asia. *JAMA Netw Open*. Published April 21, 2023.
doi:10.1001/jamanetworkopen.2023.9515

Data
Data available: No

Additional Information
Explanation for why data not available: The original data belongs to NHIRD.
